# A novel bedside cardiopulmonary physical diagnosis curriculum for internal medicine postgraduate training

**DOI:** 10.1186/s12909-017-1020-2

**Published:** 2017-10-06

**Authors:** Brian Thomas Garibaldi, Timothy Niessen, Allan Charles Gelber, Bennett Clark, Yizhen Lee, Jose Alejandro Madrazo, Reza Sedighi Manesh, Ariella Apfel, Brandyn D. Lau, Gigi Liu, Jenna VanLiere Canzoniero, C. John Sperati, Hsin-Chieh Yeh, Daniel J. Brotman, Thomas A. Traill, Danelle Cayea, Samuel C. Durso, Rosalyn W. Stewart, Mary C. Corretti, Edward K. Kasper, Sanjay V. Desai

**Affiliations:** 1 Division of Pulmonary and Critical Care, Johns Hopkins University School of Medicine, 1830 East Monument Street, Baltimore, MD 21287 USA; 2 Department of Medicine, Johns Hopkins University School of Medicine, 600 North Wolfe Street, Baltimore, MD 21287 USA; 3 Division of Rheumatology, Johns Hopkins University School of Medicine, 5200 Eastern Avenue, Baltimore, MD 21224 USA; 4 Division of Cardiology, Johns Hopkins University School of Medicine, 600 North Wolfe Street, Baltimore, MD 21287 USA; 5 Department of General Internal Medicine, 2024 E Monument St, Baltimore, MD 21205 USA; 6 Department of Surgery, Johns Hopkins University School of Medicine, 600 North Wolfe Street, Baltimore, MD 21287 USA; 7 Division of Nephrology, Johns Hopkins University School of Medicine, 600 North Wolfe Street, Baltimore, MD 21287 USA; 8 Division of Geriatric Medicine, Johns Hopkins University School of Medicine, 5200 Eastern Avenue 7th Floor, Baltimore, MD 21224 USA

**Keywords:** Medical education, Physical examination skills, Cardiopulmonary exam, Bedside medicine

## Abstract

**Background:**

Physicians spend less time at the bedside in the modern hospital setting which has contributed to a decline in physical diagnosis, and in particular, cardiopulmonary examination skills. This trend may be a source of diagnostic error and threatens to erode the patient-physician relationship. We created a new bedside cardiopulmonary physical diagnosis curriculum and assessed its effects on post-graduate year-1 (PGY-1; interns) attitudes, confidence and skill.

**Methods:**

One hundred five internal medicine interns in a large U.S. internal medicine residency program participated in the Advancing Bedside Cardiopulmonary Examination Skills (ACE) curriculum while rotating on a general medicine inpatient service between 2015 and 2017. Teaching sessions included exam demonstrations using healthy volunteers and real patients, imaging didactics, computer learning/high-fidelity simulation, and bedside teaching with experienced clinicians. Primary outcomes were attitudes, confidence and skill in the cardiopulmonary physical exam as determined by a self-assessment survey, and a validated online cardiovascular examination (CE).

**Results:**

Interns who participated in ACE (ACE interns) by mid-year more strongly agreed they had received adequate training in the cardiopulmonary exam compared with non-ACE interns. ACE interns were more confident than non-ACE interns in performing a cardiac exam, assessing the jugular venous pressure, distinguishing ‘a’ from ‘v’ waves, and classifying systolic murmurs as crescendo-decrescendo or holosystolic. Only ACE interns had a significant improvement in score on the mid-year CE.

**Conclusions:**

A comprehensive bedside cardiopulmonary physical diagnosis curriculum improved trainee attitudes, confidence and skill in the cardiopulmonary examination. These results provide an opportunity to re-examine the way physical examination is taught and assessed in residency training programs.

## Background

Sir William Osler stated that *“*Medicine is learned by the bedside and not in the classroom.” [1] However, this tenet is being challenged in the modern hospital. The time that residents spend in direct contact with patients has decreased from over 20% of their workday in the 1990’s to less than to 10% in recent years [2–5]. Many factors contribute to this shift away from the bedside, including the electronic health record (EHR), duty hour regulations, and operational pressures in academic medical centers [6–12]. It is not surprising that less time at the bedside has contributed to a measurable decline in physical exam skills [13–17], in part due to a decreased emphasis on physical diagnosis teaching and practice [7, 15, 18, 19]. Alarmingly, some studies have shown that physical exam skills, particularly cardiovascular exams skills, peak during medical school and decline during residency and beyond [20–22]. Physical exam findings directly and immediately affect patient outcomes; a decline in exam skills could have adverse effects on patient care [23, 24]. In addition to its enduring importance in patient care, the physical exam is a ritual which plays an integral role in developing a meaningful and therapeutic relationship with a patient [25, 26]. This relationship is threatened by less time and lack of emphasis on the bedside encounter [10].

The usual approach to teaching the physical exam involves an introduction to basic techniques during the first two years of medical school, followed by more focused bedside experiences during clinical years. The United States Medical Licensing Examination Clinical Skills (USMLE-CS) examination requires medical students to examine standardized patients but does not directly assess their ability to correctly identify abnormalities on real patients [27]. There is no standardized curriculum or formal assessment of physical examination skills mandated by the Accreditation Council for Graduate Medical Education (ACGME) for US residency training programs. Many internal medicine residency programs do not have physical examination curricula, and instead rely on individual attendings to provide instruction in physical exam technique and interpretation, leading to wide variability in trainee experience.

Given the high prevalence of cardiopulmonary disease in United States hospitals [28], improving cardiopulmonary exam skills among trainees has the potential to meaningfully impact a large number of patients. Cardiovascular physical exam skills peak during medical school and decline thereafter in non-cardiologists [20–22], providing an important opportunity for an educational intervention. The curriculum Advancing Bedside Cardiopulmonary Examination Skills (ACE), was developed to improve the cardiopulmonary physical diagnosis skills of trainees in a large internal medicine training program.

## Methods

### Physical examination instruction for residents prior to ACE

Before the introduction of ACE, there was no formal physical diagnosis curriculum for internal medicine residents in our program. There are several activities in which residents are exposed to physical diagnosis teaching, most notably during teaching rounds which occurs with an attending physician every morning, and during weekly activities with assigned faculty members. However, this experience is limited by the preferences of the attending and the findings of patients who are admitted to the service. There was no standardized approach to ensure that all residents received instruction in the same techniques and were able to accurately elicit and interpret physical exam findings.

### Curriculum development of ACE

ACE was designed using a formal curriculum development process for medical education [29]. The goals of ACE are to increase trainees’ appreciation for the importance of time spent at the bedside and to improve trainees’ use of the physical exam to diagnose cardiopulmonary disease. Table 1 lists the objectives of ACE. Objectives 1–3 are the focus of the current manuscript. All interns were invited to participate in ACE, including 53 from July 2015–June 2016, and 52 from July 2016–June 2017. 81.1% were in the categorical program (i.e. the standard 3-year internal medicine program), with the remainder in preliminary, primary care or combined medicine-pediatrics programs.

**Table 1:** Objectives of the ACE curriculum

After participating in the ACE curriculum, learners will:
1. Demonstrate improved understanding of the relationship between cardiopulmonary physical exam findings and physiology by achieving a higher post-ACE score on a validated cardiovascular assessment. 2. Demonstrate improved accuracy in the detection of cardiopulmonary exam findings by achieving a higher score on a validated online assessment that is administered pre- and post-ACE. 3. Demonstrate increased confidence and an increased appreciation for the importance of the bedside physical examination in patient evaluation as measured by higher scores on a self-assessment survey administered pre- and post-ACE. 4. Illustrate proper cardiopulmonary exam techniques on a cardiac simulator, healthy volunteers and hospitalized patients while being observed by a faculty preceptor. 5. Demonstrate more cost-effective use of chest radiography and echocardiography as measured by reduced ordering of inpatient computerized tomography (CT) scans and echocardiograms.

### Educational delivery methods

Interns participated in ACE as they rotated through a general medicine service at The Johns Hopkins Hospital in Baltimore, Maryland. The service is staffed by one hospitalist attending, two PGY-2s and four interns. All interns rotated on the service in either one four-week block or two separate two-week blocks. The ACE curriculum was delivered during four 30-min morning teaching sessions each week during the rotation. On average, ACE interns received a total of eight hours of dedicated physical exam instruction over 16 sessions. Approximately 75% of those sessions were at the bedside of hospitalized patients with physical exam findings. Standardized patients were not used in the ACE curriculum. ACE sessions did not replace other program-based teaching sessions, such as daily noon conferences and intern report. Six experienced faculty in Pulmonary, Cardiology and General Internal Medicine facilitated the majority of the sessions. Learning activities included:

1. *Introduction to the Cardiac Exam:* Review of basic skills on a healthy volunteer or inpatient.
2. *Introduction to the Pulmonary Exam:* Review of basic skills on a healthy volunteer or inpatient.
3. *Bedside Cardiopulmonary Physical Diagnosis Sessions:* Review of physical exam manueuvers, exam findings and their relevance to patient care using hospitalized inpatients.
4. *Physical Diagnosis and Echocardiography:* Review of echocardiographic findings of a patient examined during a previous bedside session, emphasizing physiologic correlations, utility and limitations.
5. *Mornings with the Masters:* Bedside sessions with specialists from Pulmonary Medicine, Rheumatology, Nephrology, Geriatrics, General Internal Medicine, Neurology and Infectious Disease.
6. *Interactive Computer Learning and High-Fidelity Simulation:* Interactive session using the Harvey mannequin (Laredo, Wappinger Falls, NY) or an online cardiovascular module (Blaufuss, Rolling Hills Estates, CA).

Table 2 outlines a typical 2-week ACE schedule. Subsequent 2 week blocks replaced the introductory sessions with additional bedside cardiac and pulmonary sessions, depending on the learners present. In addition, learners accessed optional online materials including an interactive tutorial and cases from Blaufuss.^10;11;22^ Supplemental optional readings emphasized proper technique, the evidence behind maneuvers, and their relationship to physiology [30, 31].

**Table 2:** Typical 2-week ACE Schedule

	Monday	Tuesday	Wednesday	Thursday
*Week 1*	Introduction to the Cardiac Exam	Introduction to the Pulmonary Exam	Mornings with the Masters	The Jugular Venous Pulse Exam
*Week 2*	Bedside Cardiac Exam	Echocardiography Session or Cardiac Simulation	Mornings with the Masters	Bedside Pulmonary Exam

### Survey instrument

The authors searched the literature and were unable to find an existing attitudinal instrument regarding the cardiopulmonary exam. A 14-item survey was designed to assess attitudes and confidence surrounding the cardiopulmonary exam. A team of content experts reviewed the final instrument to enhance content validity (BG, SD, EK, MC). Each item used a 5-point Likert scale to rate agreement or disagreement with a statement about the cardiopulmonary examination. The survey was administered to interns prior to the start of the 2015–2016 and 2016–2017 academic years, and to PGY-2s who had just completed their 2014–2015 intern year. The survey was re-administered to interns halfway through intern year in 2015–2016 and 2016–2017.

### Cardiovascular skills assessment

The Blaufuss Cardiovascular Examination (CE) consists of 50 questions divided into four categories: physiology, auditory, visual and integration. Questions contain recordings of heart sounds as well as videos of the neck and precordium. Blaufuss developed the assessment by reviewing a 1993 published survey of internal medicine (IM) program directors and Accreditation Council for Graduate Medical Education requirements for IM and cardiology fellowship training. The assessment was reviewed and modified by six academic cardiologists. It has been delivered to over one thousand medical students, graduate trainees, and practicing physicians as a measure of cardiac exam skill. In general, performance on the assessment peaks during the third year of medical school, compared to other medical school years, internal medicine residency and general practice. Only cardiology fellows and cardiology faculty outperform other groups on the assessment [20–22]. Incoming interns from 2015–2016 and 2016–2017 took the CE two weeks prior to the start of intern year and at the midpoint of the year. PGY-2s took the CE within two weeks of completion of their 2014–2015 intern year.

### Statistical analysis

Survey responses were compared using Mann-Whitney rank sum tests and Kruskal-Wallis one-way analysis of variance on ranks using the Likert scale median response. Dunn’s method was used for pairwise comparisons. CE data was analyzed using Mann-Whitney rank sum tests for intern and PGY-2 results and paired t-tests for intern mid-year CE assessments (where pre- and post-tests were available). Multilinear models stratified by exposure to ACE were run to compare overall test scores (post vs. pre) for each strata while adjusting for intern year, designation, and total pre-score. Generalized estimating equations were used to account for the repeated measures in the data (pre- and post-test score for each observation). Mutivariate linear regression was used to examine the effect of individual variables such as pre-test score, categorical versus other intern status, year of internship, gender, weeks participating in ACE, and weeks on ICU services on change in CE score at the midpoint of the year. A final adjusted model was fit including covariates that were significant at the alpha = 0.5 level in crude models. Hopkins residents’ pre-ACE performance on the CE was also compared to data reported in the literature on internal medicine residents who took the CE assessment [20, 22], using independent samples t-tests with pooled variances. A *p*-value less than 0.05 was considered statistically significant for all comparisons. Analyses were conducted using Sigmaplot (Systat Software, San Jose, CA) and SAS (SAS Institute, Inc., Cary, NC).

## Results

### Baseline assessment of interns and PGY-2s

All 53 interns from 2015–2016 (100%), all 52 interns from 2016–2017 (100%), and 29 PGY-2s from 2015–2016 (60.4%) who had not previously participated in ACE completed the survey. Results are shown in Table 3. Interns and PGY-2s “strongly agreed” that the cardiopulmonary exam is an important part of patient assessment and that improving exam skills is an important goal for the next year of training. Both groups “somewhat agreed” they had received adequate training in the cardiopulmonary exam. Compared to the interns, PGY-2s felt more confident in their ability to distinguish systolic from diastolic murmurs (*p* = 0.023) and to characterize a systolic murmur as holosystolic or crescendo-decrescendo (*p* = 0.01). PGY-2s were also more comfortable with the jugular venous pressure (JVP) examination (*p* < 0.001), and in distinguishing ‘a’ waves from ‘v’ waves (*p* < 0.001).

**Table 3:** Self-Assessment survey comparing PGY-1s who were about to start intern year, and PGY-2s who had just completed intern year

Statement	Group	Mean (SD)	Median (IQR)	*p*-value
The cardiopulmonary examination is an important part of patient assessment.	Intern	4.832 (0.511)	5.0 (5.0,5.0)	0.671
	PGY-2	4.828 (0.384)	5.0 (5.0,5.0)	
I have received adequate training in the cardiopulmonary examination.	Intern	3.743 (0.820)	4.0 (3.0,4.0)	0.884
	PGY-2	3.724 (0.751)	4.0 (4.0,4.0)	
The cardiac exam is less important now that echocardiography is widely available.	Intern	2.426 (1.228)	2.0 (1.0,4.0)	0.833
	PGY-2	2.517 (1.379)	2.0 (1.0,4.0)	
The pulmonary exam is less important now that CT imaging is widely available.	Intern	2.099 (1.162)	2.0 (1.0,3.0)	0.151
	PGY-2	2.414 (1.181)	2.0 (1.75,3.0)	
I am confident in my ability to perform a thorough pulmonary examination.	Intern	3.356 (1.006)	4.0 (2.75,4.0)	0.983
	PGY-2	3.414 (0.867)	4.0 (3.0,4.0)	
I am confident in my ability to perform a thorough cardiac examination.	Intern	3.317 (1.019)	4.0 (2.0,4.0)	0.190
	PGY-2	3.621 (0.775)	4.0 (3.0,4.0)	
I can reliably distinguish a systolic from a diastolic murmur.	Intern	3.337 (1.098)	4.0 (2.0,4.0)	0.023*
	PGY-2	3.862 (0.743)	4.0 (4.0,4.0)	
I can reliably distinguish a holosystolic from a crescendo-decrescendo systolic murmur	Intern	2.842 (1.198)	3.0 (2.0,4.0)	0.010*
	PGY-2	3.483 (1.022)	4.0 (3.0,4.0)	
I am able to distinguish a pleural effusion from a dense consolidation	Intern	2.554 (1.109)	2.0 (2.0,4.0)	0.077
	PGY-2	2.966 (1.085)	3.0 (2.0,4.0)	
I feel comfortable palpating the point of maximal impulse.	Intern	3.485 (1.205)	4.0 (2.75,4.0)	0.871
	PGY-2	3.448 (1.183)	4.0 (2.0,4.0)	
I feel comfortable assessing the jugular venous pressure.	Intern	2.950 (1.143)	3.0 (2.0,4.0)	<0.001*
	PGY-2	3.828 (0.848)	4.0 (3.0,4.0)	
I am able to distinguish “a” from “v” waves on a jugular venous pressure examination.	Intern	1.663 (0.828)	1.0 (1.0,2.0)	<0.001*
	PGY-2	2.655 (1.078)	3.0 (2.0,3.25)	
The make and model of a stethoscope is an important part of the cardiopulmonary exam.	Intern	3.158 (1.111)	3.0 (2.0,4.0)	0.709
	PGY-2	3.241 (0.830)	3.0 (2.75, 4.0)	
Improving my physical exam skills is an important goal for the next year of my training.	Intern	4.822 (0.456)	5.0 (5.0,5.0)	0.164
	PGY-2	4.724 (0.455)	5.0 (4.0,5.0)	

*n* = 101 for interns; *n* = 29 for PGY-2s. Data analyzed using Mann-Whitney U-test (*PGY* post-graduate year, *SD* standard deviation, *IQR* interquartile range)

*indicates significant difference between intern and PGY-2 using a Mann-Whitney rank sum test

**Fig. 1:**
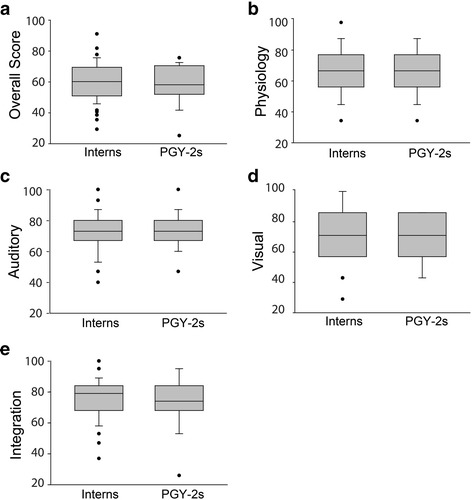
Pre-year cardiovascular examination (CE) results for interns and PGY-2s. **a** Overall scores for interns and PGY-2s, **b** Physiology scores for interns and PGY-2s, **c** Auditory scores for interns and PGY-2s, **d** Visual scores for interns and PGY-2s, **e** Integration scores for interns and PGY-2s (*n*=100 for interns, 21 for PGY-2s; PGY-2 post-graduate year 2)

52 interns from 2015–2016 (98%), 48 interns from 2016–2017 (92%) and 21 PGY-2s from 2015–2016 (44%) completed the cardiopulmonary examination (CE). Intern and PGY-2 scores were similar overall, and in all individual categories (See Fig. 1). There was no significant difference between intern and PGY-2 overall scores in our program compared to 451 internal medicine residents for whom scores were available in the literature (see Table 4) [20, 22].

**Table 4:** Comparison of Historical CE Performance and JHH Resident CE Performance

	Number of Residents	Mean Pre-test Score	Standard Deviation	t- statistic	P-Value
JHH Residents	118	60.59	11.35	.596	.5516
2006 Paper [20]	225	61.49	14.24		
JHH Residents	118	60.59	11.35	.621	.5350
2010 Paper [22]	226	61.5	13.6		

### Mid-year assessment of PGY-1s

The mid-year self-assessment survey was completed by 38 interns from 2015–2016 (72.0%) and 19 interns from 2016–2017 (36.5%) (see Table 5). At mid-year, 36 (63.2%) had participated in ACE (“ACE interns”), and 21 (36.8%) had not (“non-ACE interns”). Compared to non-ACE interns, ACE interns more strongly agreed that they had received adequate training in the cardiopulmonary exam (*p* = 0.001). Non-ACE interns agreed less strongly with this statement at the midpoint compared to the beginning of the year (*p* = 0.001).

**Table 5:** Mid-year Self-Assessment Survey Comparing ACE Interns to Non-ACE Interns

Statement	Group	Mean (SD)	Median (IQR)	*p*-value
The cardiopulmonary examination is an important part of patient assessment.	Pre-year	4.832 (0.511)	5.0 (5.0,5.0)	0.538
	Non-ACE	4.762 (0.436)	5.0 (4.75,5.0)	
	ACE	4.838 (0.374)	5.0 (5.0,5.0)	
I have received adequate training in the cardiopulmonary examination.	Pre-year	3.743 (0.820)	4.0 (3.0,4.0)	***0.001*** ^†***,***¶^
	Non-ACE	3.000 (1.140)	3.0 (2.0,4.0)	
	ACE	3.936 (0.970)	4.0 (4.0,4.25)	
The cardiac exam is less important now that echocardiography is widely available.	Pre-year	2.426 (1.228)	2.0 (1.0,4.0)	0.295
	Non-ACE	2.857 (1.493)	3.0 (1.75,4.00)	
	ACE	2.649 (0.978)	3.0 (2.0,3.25)	
The pulmonary exam is less important now that CT imaging is widely available.	Pre-year	2.099 (1.162)	2.0 (1.0,3.0)	0.152
	Non-ACE	2.238 (1.338)	2.0 (1.0,2.5)	
	ACE	2.486 (1.193)	2.0 (2.0,3.0)	
I am confident in my ability to perform a thorough pulmonary examination.	Pre-year	3.356 (1.006)	4.0 (2.75,4.0)	0.060
	Non-ACE	3.190 (1.078)	3.0 (2.75,4.0)	
	ACE	3.757 (0.723)	4.0 (3.75,4.0)	
I am confident in my ability to perform a thorough cardiac examination.	Pre-year	3.317 (1.019)	4.0 (2.0,4.0)	***0.039***
	Non-ACE	2.857 (1.108)	3.0 (2.0,4.0)	
	ACE	3.568 (0.867)	4.0 (3.0,4.0)	
I can reliably distinguish a systolic from a diastolic murmur.	Pre-year	3.337 (1.098)	4.0 (2.0,4.0)	0.854
	Non-ACE	3.381 (1.203)	4.0 (2.0,4.0)	
	ACE	3.459 (1.043)	4.0 (2.75,4.0)	
I can reliably distinguish a holosystolic from a crescendo-decrescendo systolic murmur	Pre-year	2.842 (1.198)	3.0 (2.0,4.0)	***0.022*** ^†^
	Non-ACE	2.524 (1.470)	2.0 (1.0,4.0)	
	ACE	3.378 (1.037)	4.0 (2.75,4.0)	
I am able to distinguish a pleural effusion from a dense consolidation	Pre-year	2.554 (1.109)	2.0 (2.0,4.0)	***0.048***
	Non-ACE	2.667 (1.426)	3.0 (1.0,4.0)	
	ACE	3.108 (1.149)	3.0 (2.0,4.0)	
I feel comfortable palpating the point of maximal impulse.	Pre-year	3.485 (1.205)	4.0 (2.75,4.0)	0.458
	Non-ACE	3.714 (1.146)	4.0 (3.0,4.25)	
	ACE	3.757 (1.038)	4.0 (3.0,4.25)	
I feel comfortable assessing the jugular venous pressure.	Pre-year	2.950 (1.143)	3.0 (2.0,4.0)	***<0.001*** ^‡^
	Non-ACE	3.333 (1.354)	4.0 (2.0,4.0)	
	ACE	3.757 (0.863)	4.0 (4.0,4.0)	
I am able to distinguish “a” from “v” waves on a jugular venous pressure examination.	Pre-year	1.663 (0.828)	1.0 (1.0,2.0)	***<0.001*** ^‡^
	Non-ACE	1.952 (1.117)	2.0 (1.0,2.25)	
	ACE	2.568 (1.068)	3.0 (2.0,3.25)	
The make and model of a stethoscope is an important part of the cardiopulmonary examination.	Pre-year	3.158 (1.111)	3.0 (2.0,4.0)	0.635
	Non-ACE	3.000 (1.049)	3.0 (2.75,3.25)	
	ACE	3.270 (1.217)	3.0 (2.0,4.0)	
Improving my physical examination skills is an important goal for the next year of my training.	Pre-year	4.822 (0.456)	5.0 (5.0,5.0)	0.068
	Non-ACE	4.619 (0.498)	5.0 (4.0,5.0)	
	ACE	4.757 (0.435)	5.0 (4.75,5.0)	

All participants were PGY-1s. *n* = 105 for ‘Pre’, *n* = 21 for ‘no-ACE’, *n* = 37 for ‘ACE’. Results analyzed using ANOVA on ranks (*SD* standard deviation, *IQR* interquartile range). ^†^indicates significant pairwise comparison between ‘ACE’ and ‘non-ACE’, ^‡^indicates significant pairwise comparison between ‘Pre’ and ‘ACE’, ^¶^indicates significant pairwise comparison between ‘Pre’ and ‘non-ACE’ (*ACE* Advancing Bedside Cardiopulmonary Examination Skills, *SD* standard deviation, *IQR* interquartile range)

ACE interns were more confident in their ability to perform a cardiac exam (*p* = 0.039), assess the JVP (p < 0.001), distinguish ‘a’ waves from ‘v’ waves (p < 0.001), and classify systolic murmurs as holosystolic or crescendo-decrescendo (*p* = 0.022). ACE interns felt more confident in their ability to distinguish a pleural effusion from consolidation on exam (*p* = 0.048).

**Fig. 2:**
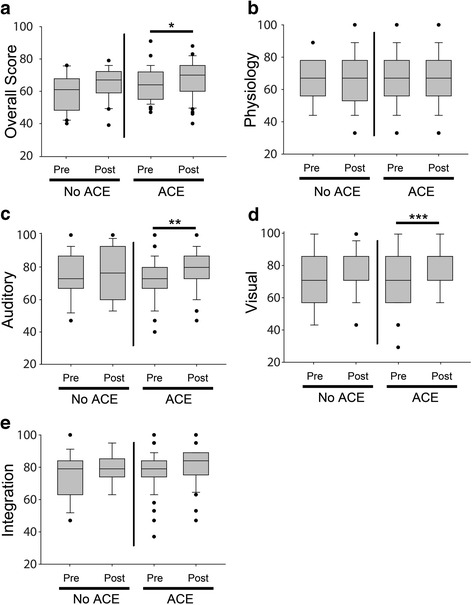
Mid-year cardiovascular assessment results compared to pre-year results. **a** Mid-year and pre-year Overall scores grouped by exposure to ACE, **b** Mid-year and pre-year Physiology scores grouped by exposure to ACE, **c** Mid-year and pre-year Auditory scores grouped by exposure to ACE, **d** Mid-year and pre-year Visual scores grouped by exposure to ACE, **e** Mid-year and pre-year Integration scores grouped by exposure to ACE (*n*=51 for “ACE” and 20 for “non-ACE”, ACE=Advancing Bedside Cardiopulmonary Examination Skills, *p=0.002, **p=0.011, ***p=0.012)

38 interns from 2015–2016 (72.0%) and 36 interns from 2016–2017 (69%) completed the CE at the midpoint of the year. 71 had completed the pre-year assessment and were included in paired analyses (see Table 6 for demographic data of mid-year participants). A total of 51 interns had rotated through ACE by the mid-point assessment compared to 20 who had not yet rotated through ACE. Results are shown in Fig. 2. Overall, interns scored significantly higher on the mid-year CE compared to their pre-year performance (intern mid-year mean 67.0 [SD 10.84], intern pre-year mean 62.42 [SD 10.31], *p* = 0.002). This difference was accompanied by an increase in auditory scores (intern mid-year auditory mean 78.62 [SD 13.27], intern pre-year auditory mean 73.96 [SD 12.81], *p* = 0.011). The intern mid-year overall score was also significantly higher than the pre-year PGY-2 performance (intern mid-year mean for all tests, 66.74 [SD 10.77], PGY-2 pre-year mean 59.47 [SD 11.95], *p* = 0.012). Intern mid-year visual scores and integration scores were also significantly higher than PGY-2 pre-year scores (Intern mean for all tests 80.28 [SD 13.47], PGY-2 pre-year mean 74[SD 17.32], *p* = 0.004 for visual scores; intern mid-year mean for all tests 80.58 [SD 10.58], PGY-2 pre-year mean 76.60 [SD 12.40], *p* = 0.026 for integration scores).

**Table 6:** Demographics of interns who participated in the mid-year assessment

	ACE	Non-ACE	
Total number of interns	51	20	
Intern Year 2015–2016	24 (47%)	7 (33.3%)	NS
Intern Year 2016–2017	27 (53%)	14 (66.7%)	NS
Female	15 (29%)	9 (43%)	NS
Male	36 (71%)	12 (57%)	NS
Categorical Program	45 (88%)	13 (65%)	*p = 0.05*
Non-Categorical Program	6 (12%)	7 (35%)	*p = 0.05*

*ACE* Advancing Bedside Cardiopulmonary Examination Skills

ACE interns’ mid-year overall scores were significantly higher than PGY-2 pre-year scores (ACE mid-year overall mean 67.71 [SD 11.04], PGY-2 pre-year overall mean 59.47 [SD 11.95], *p* = 0.027). Non-ACE mid-year overall scores were not significantly higher than PGY-2 pre-year scores (non-ACE mid-year overall mean 64.45 [SD 9.97], PGY-2 pre-year overall mean 59.47 [SD 11.95], *p* = 0.186). In paired analyses using data from interns who completed the pre- and mid-year assessments, only ACE interns had a significant increase in their overall score at the mid-year assessment (ACE mid-year overall mean 67.71 [SD 11.04], ACE pre-year overall mean 60.90 [SD 11.38], *p* = 0.019). ACE interns also had a significant increase in auditory and visual scores (ACE mid-year auditory mean 78.65 [SD 12.31], ACE pre-year auditory mean 72.67 [SD 12.45], *p* = 0.014; ACE mid-year visual mean 82.17 [SD 13.08], ACE pre-year visual mean 73.78 [SD 16.87], *p* = 0.034).

In a multilinear model using generalized estimating equations, ACE interns had post-ACE scores that were on average 4.98 points higher than their pre-ACE score (*p* = 0.001). Holding all other factors constant, ACE interns in the second year of the curriculum had scores that were on average 2.88 points lower than interns who participated in the first year (*p* = 0.0077). ACE categorical interns had scores that were on average 5.95 points higher than non-categorical interns (*p* = 0.0098). ACE pre-score was a significant predictor of post-test score (*p* < 0.001). Non-ACE interns had post-test scores that were on average 5.4 points higher than their pre-year scores, but this difference was not significant. Non-ACE pre-score was a significant predictor of post-test score (See Table 7).

**Table 7:** Multilinear Models with Generalized Estimating Equations, Stratified by Exposure to the ACE curriculum

	Estimate	*P*-Value
Non-ACE Interns		
Test		
Post	5.41	0.0633
Pre = Ref	–	–
Intern Year		
Year 2	2.41	0.3037
Year 1 = Ref	–	–
Designation		
Categorical	2.85	0.2805
Not Categorical = Ref	–	–
Total Pre Score	.585	*<0.0001*
ACE Interns		
Test		
Post	4.9852	*0.0012*
Pre = Ref	–	–
Intern Year		
Year 2	−2.8754	*0.0077*
Year 1 = Ref	–	–
Designation		
Categorical	4.9413	*0.0098*
Not Categorical = Ref	–	–
Total Pre Score	0.7750	*<0.0001*

For each condition, ACE and non-ACE, a multilinear model was generated using generalized estimating equations to predict the change in post-test score for each variable while holding the others constant (*ACE* Advancing Bedside Cardiopulmonary Examination Skills Ref = reference)

In an adjusted multivariate linear regression model, two variables significantly predicted overall change in score for all interns: pre-test score and categorical status. The difference between the pre and post-test scores became smaller for every unit increase in pre-test score (*p* < 0.001). Categorical interns had a change in score that was on average 7.61 points greater that non-categorical interns (*p* = 0.0185). Participation in the ACE curriculum was not a significant predictor of change in score in this model (see Table 8).

**Table 8:** Multivariate Linear Regression Model

	Adjusted Estimates	P-Value
Any ACE (Yes)	−.373	.8945
Year		
Intern Year 1 (Reference)		
Intern Year 2	−4.27	.0781
Designation		
Not Categorical (Reference)		
Categorical	7.61	*.0185*
Total Pre-test Score	−.614	*<0.0001*

In this multivariate linear regression model, change in test score is the dependent variable, ACE is the exposure, and intern year, categorical vs. non-categorical, and pre-test score are the covariates (*ACE* Advancing Bedside Cardiopulmonary Examination Skills)

## Discussion

In the current academic medical center, many factors pull physicians, particularly trainees, away from the bedside. As physicians spend less time with patients, opportunities to practice and teach core skills such as the physical exam have declined [32]. This has contributed to a decline in skill among both trainees and practitioners, and contributes to diagnostic error [24]. While technological advances have dramatically improved our ability to diagnose disease, the physical exam still outperforms technology in a number of important instances [33]. In addition to its diagnostic importance, the physical exam is a vital component of the patient-physician relationship [10]. It helps to build rapport and trust, and if performed properly, can even contribute to a patient’s overall sense of well-being. Spending time at the bedside of patients also provides meaning for a physician’s work. The alarming rise in physician burnout in recent years may partly reflect this shift away from the bedside [34, 35]. There is a growing international movement to bring physicians and trainees back to the bedside [36]. The present study is a direct outgrowth of that movement, and supports the hypothesis that a dedicated curriculum coupled with regular practice improves physical diagnosis skills.

It was encouraging that both interns and PGY-2s strongly agreed that improving their physical examination skills was an important goal for the next year of their training. This suggests that the introduction of the ACE curriculum was a timely intervention for the residency program. Interestingly, interns and PGY-2s reported a similar level of confidence in their ability to perform a cardiac and pulmonary exam, but reported differences in confidence surrounding specific maneuvers such as auscultation of murmurs and the jugular venous pulse examination. The fact that PGY-2s in this study were more confident on certain exam skills than incoming interns but performed at the same level on the CE might reflect a lack of emphasis on physical diagnosis teaching and practice prior to the start of the ACE curriculum. This gap between confidence and competence is an important one to address for trainees, as it directly impacts the safety and quality of patient care [37, 38].

Interns who participated in ACE had improved confidence in their physical exam skills. Interns who had not yet participated in ACE had diminished confidence compared to their peers but also compared to the start of internship. This might reflect a realization that non-ACE interns had not received adequate physical examination training to that point in their careers. The areas in which confidence differed between interns and PGY-2s as well as between ACE and non-ACE interns tended to be areas of the exam that are more technically challenging (e.g. distinguishing ‘a’ waves from ‘v’ waves). This might provide some insight into the design of future curricula that focus on more difficult exam maneuvers and techniques.

The finding that ACE interns had a significant improvement in their mid-year CE scores provides evidence that the curriculum was effective in improving exam skills. The fact that ACE interns had a significant improvement in their mid-year CE scores compared to PGY-2s who had completed intern year prior to ACE, further suggests that the ACE curriculum was more effective than the previous approach to physical exam teaching in our residency program. Since most training programs utilize the “traditional” approach to physical exam teaching, this could inform the development of similar curricula at other institutions.

In the stratified multilinear models with generalized estimating equations, only ACE interns had a significant increase in their post-test scores. However, in our regression model that combined both ACE and non-ACE interns, ACE participation did not significantly predict change in CE score. Since this was a not a randomized trial, but a quasi-randomized study based on pre-determined intern schedules, other factors may have limited our ability to see a significant effect of the ACE curriculum in the final regression model. It is not surprising that categorical status predicted a higher change in score, since categorical interns spend more time on the internal medicine services during their intern year. It is also not surprising that pre-year score was a predictor of change in score. Lower performers at the outset had more room to improve compared to those interns with a higher pre-year score.

This study has several limitations. This was a single center experience so the results may not be applicable to other institutions. However, the fact that interns and residents from our program performed similarly to other internal medicine residents as reported in the literature before exposure to ACE, suggests that our trainees were starting from a similar knowledge and skill level before introduction to the curriculum.

Our survey, while developed and reviewed by faculty who are experts in the physical exam, has only been used on a single population and lacks robust validity evidence. It was not possible to correlate individual responses with CE performance as the survey was anonymous.

We captured nearly 100% of the interns on the initial assessments, but only 60% and 44% of PGY-2s from 2015–2016 participated in the survey and CE, respectively. We also lost several interns at the mid-year assessments since their clinical schedules made it difficult for some interns to participate. This may have led to our final analyses being underpowered to detect differences in overall scores, particularly for the non-ACE interns. Non-categorical residents were over-represented in the non-ACE population at the mid-year point of the year, which may have also affected our results.

Interns and teaching attendings rotate with one another on other services. It is likely that exam skills emphasized during the general medicine rotation where ACE is delivered were taught to non-ACE interns by faculty and peers. This spillover effect probably increased intern skills independent of ACE participation, and is a recognized benefit of the current residency training model. Since the ACE curriculum was not blinded to participants, it is possible that the survey results were biased by the expectation that participation in a dedicated physical exam curriculum would improve confidence and skill.

Familiarity with the Blaufuss software used during ACE may have allowed ACE interns to score higher on the mid-year assessment. Since the Blaufuss software was used only once per rotation, it seems unlikely that this played a major role in the final results. It is also possible that taking the CE a second time might have led to increased scores independent of participation in ACE. However, in previous studies using the CE, control groups did not have improvements in scores upon taking the test a second time [21].

It is possible that the improvements in ACE intern performance could have been due to other aspects of the general medicine teaching service that were not part of the ACE curriculum. Rounds on the ACE service were similar to rounds on other hospitalist-led services. Aside from the ACE curriculum, the teaching activities on that service were shared activities with other rotations such as daily noon conference and intern report. Hospitalist attendings rotated through the ACE service as well as the other hospitalist teaching services. The patient population on the ACE service was similar to other general medicine services. As a result, it seems more likely that any changes in ACE intern performance were due to the ACE curriculum.

## Conclusions

Implementation of a bedside cardiopulmonary physical diagnosis curriculum improved the attitudes, confidence and skill of interns in the cardiopulmonary examination. The effectiveness of the ACE curriculum can inform further improvements in physical exam teaching and assessment, and lay the groundwork to examine the effect of these interventions on important metrics such as test utilization, cost of care, patient and provider satisfaction, diagnostic error, and clinical outcome.

## Abbreviations

ACE: Advancing Bedside Cardiopulmonary Physical Exam Skills
ACGME: Accreditation Council for Graduate Medical Education
CE: Cardiovascular exam
CT: Computerized tomography
EHR: Electronic health record
IQR: Interquartile range
JVP: Jugular venous pressure
PGY-1: Post-graduate year 1 (intern)
PGY-2: Post-graduate year 2
SD: Standard deviation
USMLE – CS: United Stated Medical Licensing Examination – Clinical Skills
